# Cystic Fibrosis: Brazilian ENT Experience

**DOI:** 10.1155/2012/204696

**Published:** 2012-05-06

**Authors:** Tania Sih, Ricardo Godinho, Leticia Paiva Franco, Otávio Piltcher

**Affiliations:** ^1^Faculty of Medicine, Laboratório de Investigações Médicas (LIM), Number 40, Universidade de São Paulo, São Paulo, Rua Mato Grosso, 306/1511, 01239-040 São Paulo, SP, Brazil; ^2^Health and Biological Sciences Institute, Department of Medicine, Pontifical Catholic University of Minas Gerais-PUC Minas, Rua Dr Chassim 208, 35700-018 Sete Lagoas, MG, Brazil; ^3^Department of Otorhinolaryngology, School of Medicine, Universidade Federal de Minas Gerais, Avenida Alfredo Balena 180, 30000-000 Belo Horizonte, MG, Brazil; ^4^Department of Otorhinolaryngology, College of Medicine, Universidade Federal do Rio Grande do Sul, Avenida Bento Gonçalves 8083, Casa 2, 91540-000 Porto Alegre, MG, Brazil

## Abstract

Most published studies about Cystic Fibrosis (CF) are European or North American. There are still few publications about the characteristics of fibrocystic populations in developing countries. The incidence of cystic fibrosis (CF) in Brazil varies among different regions (1 : 10,000 in Minas Gerais, 1 : 9,500 in Paraná, 1 : 8,700 in Santa Catarina, and 1 : 1600 in Rio Grande do Sul). The prevalence of the DF508 mutation also varies according to population: 33% in Sao Paulo, 49% in Rio Grande do Sul, 27% in Santa Catarina, and 52% in Minas Gerais. Cough and nasal obstruction are the most common symptoms. The variation in nasal polyposis prevalence may be explained by population genotypic characteristics in a country that spans a continent. Findings on nasal endoscopy and computed tomography (CT) have better correlation than do this information compared with surgical and clinical history. Microbiologic studies suggest a high level of early contamination of the airways. Sensorineural hearing loss (SNHL) occurs in these patients as a result of ototoxic antibiotics. The data compiled in this paper is useful, but also lead to the general agreement that more research would be welcome due to the unique characteristics of this country.

## 1. Introduction

Cystic fibrosis (CF) is the most common inherited autosomal recessive disease among Caucasians [1–6]. It has a frequency of 1 : 2.000 to 1 : 6.000 in the Caucasian population of developed countries [1, 3, 4, 6–9], being rare in populations with African (1 : 30.000) and Asian (1 : 90.000) origins [10, 11]. In Brazil, the incidence is 1 : 9.500 in Paraná [12] and 1 : 8.700 in Santa Catarina [13] and 1 : 10.000 in Minas Gerais [14]. The disease is caused by mutations in the gene encoding the cystic fibrosis transmembrane regulator protein (CFTR), mapped in the human chromosome at 7q31. This gene, described in 1989 [15–17], encodes a protein that acts as a chloride channel, and its dysfunction results in an abnormal transport of sodium and chloride through the apical membrane of epithelial cells of the upper aerodigestive tract and exocrine glands. Abnormal flow of salt and water leads to dehydration of fluids of the exocrine glands and a change in the viscoelastic properties of mucins [5, 6, 18, 19].

Changing the composition and viscosity of mucus leads to dysfunctional mucociliary clearance and to obstruction of the paranasal sinus ostia, predisposing to local inflammation with consequent hypoxia and increased partial pressure of carbon dioxide. This results in mucosal edema and greater compromising of ciliary function and favors bacterial colonization and infection, particularly by *Staphylococcus aureus* and *Pseudomonas aeruginosa *[3, 6, 18–20]. Nasal polyposis in CF patients was described for the first time in 1959 [21]. Its frequency varies in different populations and depends on the evaluative technique. A compromised nasal sinus is believed to aggravate the pulmonary picture [20], and thus the participation of otolaryngologists to address this particular group of patients becomes very important. Another important issue for the otolaryngologists in monitoring CF patients is their hearing. In spite of the low prevalence of middle ear disease in these patients, there is in fact sensorineural hearing loss caused by ototoxic drugs.

Most articles on this subject are European or American, involving both children and adults with CF. There are still few publications about the characteristics of fibrocystic populations in developing countries and, in general, with small samples. In Brazil since 1972 when the first specific CF center was opened in Rio de Janeiro many centers have been developed. During the last decades more than 13 centers were created first in the southwest and south. Today more CF clinics are being recognized all over the country. As a result more national literature has been available, but there is still a need to better characterize CF patients with regard to rhinosinusitis. There is great genetic heterogeneity as well as a wide range of mutations with large variety of clinical presentations observed, which may be explained by particular phenotypic characteristics in the Brazilian population and even populations of each region in the country, since Brazil has continental dimensions and represents a unique patient population given its European and American ancestry (Table 1).

The purpose of this study is to gather data from the main Brazilian publications on otorhinolaryngological manifestations of CF patients and to compare with studies from other countries.

## 2. Symptomatology

In a Brazilian study involving 100 children and adolescents with CF in the state of Minas Gerais, the most commonly reported symptoms in addition to cough (45%) were oral breathing (44%), restless sleep (42%), nasal obstruction (37%), halitosis (33%), headache (30%), and rhinorrhea (29%). Only two patients complained of anosmia and about 10% complained of periorbital or facial pain [22]. It is important to highlight that, although some studies show that up to 100% of patients demonstrate pansinusitis on tomography of the paranasal sinuses, 20% of these patients did not present any of the symptoms listed, and 36% did not present bilateral changes in the middle meatus [22]. One possible explanation for a low frequency of chronic sinonasal complains would be a matter of priority, being pulmonary and gastrointestinal problems prioritized by patients and family. Furthermore, an “adaptation” to chronic nasal symptoms may occur [23].

Boari and Castro Júnior [24] identified the following CF symptoms whilst evaluating 34 CF patients from the city of Sao Paulo: cough (88.2%), headache or facial pain (38.2%), and nasal obstruction (29.1%). Rhinorrhea was reported by only four patients. Franche and coworkers [25] in Porto Alegre, the southeast Brazilian's capital in Rio Grande do Sul state, found only two out of 23 patients with bilateral polyposis and concerning nasal obstruction, nasal secretion, and headache only eight (34.8%) reported nasal obstruction, ten (43,5%) complained of nasal secretion, and none had headache. Weber and Ferrari [26] (Botucatu, Sao Paulo State) evaluated 23 patients with an average age of 6 years and 4 months, and 22% of them complained of oral breathing.

## 3. Endoscopic Findings

There is a great variety of endoscopic findings in CF patients and a trend toward increased incidence of diagnoses of nasal polyps over time. From 1961 to 2005, the prevalence of nasal polyposis varied from 5% to 57% [20, 24, 26–34]. This may be explained by the increased survival rate of CF patients in developed countries, by selection of patients, by size of samples, by differences of age groups studied, and mainly by the most recent routine use of nasal endoscopy as a diagnostic technique [1, 19, 20]. Boari and Castro Júnior [24] compared clinical, tomographic, and endoscopic findings and verified that nasal endoscopy contributed enormously to the evaluation of chronic rhinosinusitis and of nasal polyposis in CF patients, reliably characterizing sinonasal conditions.

Other studies carried out in Brazil have also shown considerable variation in frequency of diagnosis of nasal polyps in CF patients. Franco and colleagues [22] found nasal polyps in only 14% of children and adolescents with CF even using an evaluative technique similar to that used in other studies published recently. Weber and Ferrari [26] found nasal polyps in 39.1% of 23 CF patients aged one year and nine months to 22 years and 8 months. Boari and Castro Júnior [24] found polyps in only three (8.82%) out of 34 CF patients, from six to twenty-two years of age, evaluated using nasal endoscopy. Franche and coworkers [25] found only two out of 23 patients with bilateral nasal polyps in both cases. Sakano and colleagues [20] identified nasal polyps in 36% of 50 patients, over two years of age, evaluated using rigid endoscopy under general anesthesia, a method that may have allowed more accurate diagnosis of polyps, even small ones.

 There is much genetic heterogeneity in CF, that is, a wide range of mutations with a great variety of clinical presentations. Thus the variation found in related nasal polyposis may be explained by particular genotypic characteristics of populations from a country that spans a continent. We should also consider underdiagnosis of CF in Brazil and the premature deaths of patients with pulmonary complications and, maybe, those with associated nasal polyposis. In Brazil, the survival of CF patients is lower than that in developed countries where expectance of survival reaches 31.6 years. In a Brazilian study conducted by Alvarez and colleagues [35], the mean age of survival for 104 CF patients evaluated was 18 years and four months after diagnosis.

 Among 100 CF children and adolescents studied by Franco and coworkers [22], patients with nasal polyps had associated nasal secretions, identified using endoscopy, but endoscopy did not identify the presence of nasal polyps and symptoms such as cough, rhinorrhea, oral breathing, restless sleep, headache, and nasal obstruction. In this study, there was no statistically significant association between nasal polyps and patients' age; however, 64.3% of patients with polyps in this study by Franco et al. [22] were between four and 12 years of age, which coincides with ages in the report by Sakano and colleagues [20] (88.89% of cases of polyposis in patients under 15 years of age). That is, among Brazilian children with CF, the prevalence of polyps in younger children is quite high. Nasal polyps have been identified in a child as young as 8 months old; a differential diagnosis was made using computed tomography of the nose and paranasal sinuses [22].

Weber and Ferrari [26] did not find any association of gender, age, clinical severity, or genetic mutation with the presence of nasal polyposis. The 14 children with nasal polyps evaluated in the study conducted by Franco and coworkers [22] had maximum scores, according to the Lund-Kennedy scoring system, and polyposis obliterans were not found. Weber and Ferrari [26] classified the polyposes according to the scale by Johansson and colleagues [36]: grade I in four patients (44.4%), grade II in one patient (11.1%), and grade III in four patients (44.4%). Using endoscopy, Franche and coworkers [25] found other significant endoscopic evidence in 23 CF patients. The inferior turbinate was hypertrophic in 34.8%. In 22 out of the 46 nasal cavities examined, the mucosa of the middle turbinate was normal in 47.8%, pallid in 34.8%, and hyperemic in 17.3%. Most patients (65.2%) had secretion in the nasal cavity: serous (53.3%), mucoid (26.7%), and purulent (20%). The relationship was statistically significant between hyperemia of mucosa of the middle turbinate and presence of nasal discharge, with positive culture of aspirate from the middle meatus (*P* = 0.0085 for hyperemic mucosa and *P* = 0.00272 for nasal discharge).

Sakano and coworkers [20], using endoscopy, found secretion in the middle meatus in 54% of cases and medial bulging of the lateral nasal wall in 58%. Franco and colleagues [22] found medial bulging of the lateral nasal wall in 41% (bilateral in 36% of them) and did not find any association between age and the presence of bulging. Boari and Castro Júnior [24] evaluated results of nasal endoscopy according to the Lund-Kennedy scale [37] concerning the distribution of mucosal edema, nasal secretions, and nasal polyps and, considering criteria for the endoscopic diagnosis of chronic rhinosinusitis, found that 73.52% had the disease at the time of assessment.

## 4. Profile of Cytokines in Sinonasal Polyposis in CF

Sinonasal polyposis in CF is classified as noneosinophilic sinonasal polyposis, according to the histopathological findings. To better understand the pathogenic mechanisms of nasal polyps in CF, several studies have tried to characterize the inflammatory microenvironment, cytokines, adhesion molecules, and ionic transport. A Brazilian study conducted by Nunes and coworkers [38] analyzed the expression of messenger ribonucleic acid (mRNA) for cytokines IL4, IL5, IL6, IL8, GM-CSF, and INF-gamma in patients with CF, who had nasal polyposis, and in a control group, using reverse transcription-polymerase chain reaction (RT-PCR). The resulting expression of mRNA for IL5, IL6, IL8, and GM-CSF was similar in the group with CF polyposis and in the control group. Low values of INF-gamma and a tendency to higher values of IL4 were associated with the CF group. The reduction of INF-gama would be responsible for the presence of bacteria or bacterial LPS (lipopolysaccharides) in the intracellular medium. These bacteria stimulated production of IL4 by Th2 cells. The results would reflect intense inflammation with an increase in the eosinophilic cationic protein, total IgE, intermediated by the increase of IL4 and the low INF-gama [38].

## 5. Tomographic Findings

Computed tomography (CT) has become a valuable tool for the diagnosis and monitoring of disorders of the upper airway in CF patients and is also essential in planning surgical cases, to study osseous structures. The main manifestations are opacification of paranasal sinuses, formation of mucoceles/pseudomucoceles, agenesis or hypoplasia of the frontal sinus, and medial bulging of the lateral nasal wall [39–43]. Virtually all paranasal sinuses were found radiologically to be affected in over 90% of patients over 8 months of age [2].

Boari and Castro Júnior [24], using CT, evaluated 31 patients with CF, and found 93.54% presenting chronic rhinosinusitis, as seen on radiologic studies. The Lund-Mackay classification of tomography staging [44] had an average score of 13.3, varying from 1 to 24. The most affected sinus was the maxillary sinus, with 91.9% having opacification (45.1% complete opacity and 46.8% partial). In descending order, there was a higher incidence of involvement of the anterior ethmoid sinus (83.9%), frontal sinus (70%), sphenoid (66.7%), and posterior ethmoid (54.8%). Despite important changes on tomography, Boari and colleagues believe that sinonasal disease shown by CT does not seem to be directly related to surgical findings and that CT and nasal endoscopic examination present a higher percentage of positive correlation, while CT used with the medical history presented the lowest correlation. The same authors believe that based just on tomography there is probably an overestimation of diagnosis of sinonasal disease.

Kobayashi and colleagues compared the Lund-Mackay scores of tomography with various clinical aspects from patients in a referral center in the south of Brazil (Rio Grande do Sul) and found no statistical correlation [45]. They discussed that this score was not developed to analyze these disease, but anyway show at the same time the need for a specific tomography graduation system for CF patients and that image alterations in these patients should be really carefully evaluated as symptomatic and asymptomatic patients had similar results. Sakano and coworkers [20] examined 50 patients with CF using tomography and found absence of changes in 4%, mucosal thickening in 50%, opacification of paranasal sinuses in 6%, and pseudomucocele in 40%. No statistically significant differences were found between findings on tomography and different genotypes or patient status of health. In this study, there was an association between the presence of medial bulging of the lateral nasal wall identified using endoscopy and using CT, with 65.5% of cases having pseudomucocele.

A condition described by Coste and colleagues [39], pseudomucocele is made evident on CT scans specifically in patients with CF. Published reports show controversy concerning terminology for this pathological entity of the sinuses, more studied since routine tomographic examinations became part of the evaluation of patients with CF. Some authors use the term “mucocele” and others “mucopiosinusitis.” Most often the more accurate term is “pseudomucocele” since there is viscous secretion in the paranasal sinuses, determining CT images of central hyperdensity and peripheral hypodensity. It is not a true epithelially walled cystic lesion but rather secretion surrounded by inflammatory tissue which follows the shape of the walls of the sinuses, with a tendency to expand [39].

Portes and colleagues [46] (Sao Paulo) reported a case of a child two years and one month old with chronic nasal obstruction since birth; CT showed opacification of the maxillary and ethmoid sinuses bilaterally and the formation of a cystic appearance with a halo of peripheral enhancement around the maxillary sinuses. These cystic formations, pseudomucoceles, led to protrusion of the lateral nasal wall and narrowing of the nasal cavity. Pseudomucocele, therefore, may be considered a frequent manifestation found in studies of series of nasal manifestations in CF patients.

Thomé and coworkers [47] reported a case of a 10-month-old child with nasal obstruction, ethmoide tomographic findings compatible with mucocele and CF was confirmed by the sweat test [47]. Diagnosis of mucoceles is commonly established using computed tomography (CT) of paranasal sinuses, which shows osseous erosion of the walls of paranasal sinuses, with smooth outward displacement. Upon magnetic resonance (MRI), mucoceles will show variable signal intensities in both T1-and T2-weighted images. A disadvantage of MRI when compared with CT is the absence of osseous details; therefore MRI has no value in surgical planning. In pediatric patients with CF and suspected mucocele, MRI is essential to help eliminate other entities such as meningocele, rhabdomyosarcoma, hemangioma, and neuroblastoma [48].

## 6. Microbiology

 Only two Brazilian studies involving CF patients evaluated upper airways from a microbiological point of view. Franche et al. [25], from Porto Alegre (Rio Grande do Sul State), analyzed the bacteriology of aspirate from the middle meatus and sputum of 23 patients with CF. The Junh-Tym-Tap aspiration system and a rigid endoscope were used to collect material. There was a total of 42 aspirations. In seventeen (73.91%) out of 23 patients, cultures were negative; in six they were positive (26.08%). Out of 42 aspirations, 73.81% were negative, and 26.19% (11 aspirates) were positive. Out of 11 positive aspirations two showed *Pseudomonas aeruginosa* (both from the same patient), three showed *Staphylococos aureus*, two *Haemophylus influenzae*, two *Streptococcus penumoniae*, and two *Acinetobacter lwoffi*. Samples of sputum from all patients presented bacterial growth, 36.43% with growth of two germs, the growth of *Pseudomonas aeruginosa* in 40%. Other bacteria found in the sputum were mucoid *Pseudomonas aeruginosa* (20%), *Staphylococcus aureus* (23.33%), *Haemophilus influenzae* (6.68%), *Serratia marcescens* (3.33%), *Acinetobacter lwoffi* (3.33%), and *Streptococcus pneumoniae* (3.33%). No association was observed between the results of sputum cultures and results for aspirate from the middle meatus [24].

 Sakano and coworkers [20], from Campinas (Sao Paulo State), collected samples using a swab of the oropharynx, secretions gathered by maxillary antrostomy, and secretions gathered using endotracheal tubes, representing tracheal secretions from 50 CF patients. The distribution of results of cultures from different sites (maxillary sinus, trachea, and oropharynx) was as seen in Table 1. The incidence of colonization by *Pseudomonas aeruginosa* and *S. aureus* was 53% and 80%, respectively. Simultaneous findings of *P. aeruginosa* in three sites investigated in children and adolescents who were evaluated are directly related to pathophysiology of chronic colonization of CF patients by this bacterium and suggest a high level of early contamination of the airways.

## 7. Genetics

 The gene for CF is located in chromosome 7, at locus q31, formed by 250 kilobases of DNA, with 27 exons. It encodes an mRNA of 6.5 kilobases that transcribes a transmembrane regulator protein for ionic transport, comprising 1480 amino acids, known as CFTR (cystic fibrosis transmembrane conductance regulator). The CFTR protein is essential for ionic transport through cellular membranes, being involved in regulating the flow of Cl, Na, and water [15–17].

One of the most important aspects of the current approach to CF is the great heterogeneity of the disease regarding its presentation, clinical course, and prognosis. Since the gene for CF was discovered, many hundreds of mutations have already been identified. The most frequent mutation in Caucasians is ΔF508, deletion of three base pairs, causing the loss of an amino acid (phenylalanine) at the 508 position of the CFTR protein, which prevents its appropriate functioning. In Brazil, the prevalence of mutation in ΔF508 varies according to the population studied. It is present in 27% to 52% of chromosomes: Martins and coworkers [49] found 33% in Sao Paulo and Raskin and colleagues [50] found 49% in Rio Grande do Sul, 27% in Santa Catarina, and 52% in Sao Paulo. Alvarez and coworkers [35] genotyped 92.31% of 104 patients studied in Campinas, and the ΔF508 mutation was present in 50% of 192 chromosomes studied. A lower prevalence of this mutation is expected in populations with great ethnic diversity. Other mutations found by Alvarez and colleagues were G542X (4.17%), N1303K (2.08%), G551D (1.04%), R553X (0.52%), and W1282X (0.52%).

Weber and Ferrari [26] investigated genetic mutations of 23 patients studied, and eight patients were found with the genetic mutation ΔF508/other, three ΔF508/ΔF508, one ΔF508/G542X, one G542X/other, one R1162X/R1162X, and nine patients without specific mutations. In this study, no association was found between the genotype and the presence or severity of nasal polyposis [25].

The study conducted by Sakano and coworkers [20] researched the most frequent mutations in Brazil, dividing into three groups: ΔF508/ΔF508 (homozygotes), ΔF508 heterozygotes, and other mutations. Out of the 50 patients evaluated, results were 38%  ΔF508 heterozygotes, 32%  ΔF508 homozygotes, other mutations in 20%, and 10% were not evaluated. No association was observed between the severity of the disease and the genotype investigated. An association was observed between CF genotype with the presence of nasal polyposis (*P* = 0.006), but no association was observed among genotype and gender, age, secretion in middle meatus, or medial bulging of the lateral nasal wall. The patients with the ΔF508 heterozygote had more abnormalities seen on CT than did the homozygotic patients, but there was no statistically significant difference. Sakano's group believes the absence of an association of the genotype with several of the variables studied is due to the small size of the sample.

## 8. Otological Findings

Martins and coworkers [51], from Belo Horizonte (Minas Gerais State), evaluated 120 clinically stable patients with CF. Of these, 57% had had no otitis media in the past, 65% had had no otitis in the first year of life, and 71% had had no otitis in the year before being included in the study. One hundred and thirteen patients (94%) had normal otoscopy; only 3% had alterations indicating otitis media with effusion. Tympanometry showed a type A tympanogram in 91%.

The sensorineural hearing loss (SNHL) in CF patients may be caused by ototoxic drugs such as aminoglycosides, commonly indicated for the treatment of pulmonary diseases in CF patients. Piltcher and colleagues [52], from Porto Alegre (Rio Grande do Sul), conducted a retrospective study involving 107 medical charts of children with CF, with an average age of 7.87 years (SD 4.49). Audiological tests were carried out in only 39.3% of these children, and 28% were diagnosed with sensorineural hearing loss of varying degrees. The stapedial reflex was absent in 36% of cases. Tympanometry showed a curve A in 91%. All patients received a variable amount of aminoglycosides courses. The study was unable to correlate the amount of this antibiotics and or severity of disease with the prevalence of hearing loss. The frequent use of aminoglycosides and the relatively high prevalence of sensorineural hypoacusia reinforce the importance of an audiological evaluation of patients with CF to improve the quality of life and survival of this population in Brazil. Unpublished data from this same center (Weigert et al.) indicates that ultrafrequency audiometry would show even higher prevalence of early hearing loss detection among these patients.

## 9. Conclusion

 There has been increasing amount of data on CF patients around the world. Brazil followed this same tendency of scientific publications. What makes Brazil's data very important is its unique continental geographical characteristics and population with a colonization history that leads to a great amount of mixigenation between European and Native American ancestors. If those facts interfere with the clinical presentation of this genetic common disease is not known yet. The lower survival rates compared to developed countries do not seem to be directly related to those genetic differences, but raise the possible existence of mutations not yet known that lead to undiagnosed patients. On the other hand it is clear that the actual state of medicine practice for those patients has been improving and this results from recognition by Brazilian physicians for the need to follow international tendency and establish multidisciplinary centers to follow and control these patients. As a consequence there will be an increasing amount of research and data to be shared. The best performance is also confirmed in improving survival rates, despite the need to continue to seek medical awareness about the importance of early diagnosis. The Brazilian experience confirms, as do reports from other countries, that an otolaryngologist should be part of an interdisciplinary team, with an important role in the diagnosis and appropriate management of direct and indirect manifestations of this disease. Hearing must be followed closely due to systemic intravenous antibiotics. As in other countries sinus alterations must be also better understood as most patients have it, but few present symptoms. The upper respiratory phenotypic manifestations among Brazilians do not seem to be really different from other populations. It also brings up to discussion the need to study rhinosinusitis among these patients as a different entity. When it should be considered a disease and how to treat it remain a basic issue.

## Figures and Tables

**Table 1:** Symptoms, polyps, tomography and mutations in Brazilian CF patients.

	City: Sao Paulo City Estate: Sao Paulo Boari and Castro Júnior [24]	City: Botucatu Sao Paulo Weber and Ferrari [26]	City: Campinas Sao Paulo Sakano and colleagues [20]	City: Belo Horizonte Minas Gerais Franco and colleagues [22]	City: Porto Alegre Rio Grande do Sul Franche and colleagues [25]
Symptoms	Cough (88.2%), headache or facial pain (38.2%)	22% of them complained of oral breathing		Cough (45%), oral breathing (44%)	34.8% reported nasal obstruction, ten (43,5%) complained of nasal secretion

Polyps	8.82%	39.1%	36%	14%	8.69

Tomography	(i)Lund-Mackay average score of 13.3 (ii)considering criteria for the endoscopic diagnosis of chronic rhinosinusitis: 93.54%	According to Johansson and colleagues: grade I in four patients (44.4%), grade II in one patient (11.1%), and grade III in four patients (44.4%)	Mucosal thickening in 50%, opacification of paranasal sinuses in 6% and pseudomucocele in 40%	According to the Lund-Kennedy score: 14 children with nasal polyps had maximum scores	

Mutation in ΔF508		Out of 23 patients: 34% patients were found with the genetic mutation ΔF508/other, 13% ΔF508/ΔF508, 4% ΔF508/G542X, 4% G542X/other, 4% R1162X/R1162X, and 39% patients without specific mutations	Out of the 50 patients: 38% ΔF508 heterozygotes, 32% ΔF508 homozygotes, other mutations in 20%		
